# Association between dietary folate intake and gestational diabetes mellitus: evidence from baseline data of a Chinese maternal and infant cohort

**DOI:** 10.3389/fnut.2025.1702918

**Published:** 2025-12-03

**Authors:** Xiangyi Wu, Rong Zhang, Lu Lu, Rui Fan, Rui Hu, Yue Teng, Lina Pan, Xiaoling Zeng, Wei Jiang, Wei Li, Ling Dong, Zhaofeng Zhang, Wenli Zhu

**Affiliations:** 1 Department of Nutrition and Food Hygiene, School of Public Health, Health Science Centre, Peking University, Beijing, China; 2 Gaomi City People's Hospital, Weifang, China; 3 Beijing Huairou Maternal and Child Health Care Hospital, Beijing, China; 4 Beijing Haidian Maternal and Child Health Hospital, Beijing, China; 5 Hunan Ausnutria Institute of Food and Nutrition, Changsha, China

**Keywords:** dietary folate intake, folate, folic acid supplement, gestational diabetes mellitus, methyl-donor nutritional quality index, one-carbon metabolism, vitamin B_12_

## Abstract

**Introduction:**

Folate is an essential nutrient during pregnancy, which may contribute to the development of gestational diabetes mellitus (GDM), but the results of existing studies are inconsistent. This study aimed to assess the dietary folate intake among pregnant women and examine its association with GDM.

**Method:**

Baseline data from the Mother & Child Nutrition and Health Cohort Study (MCNHC) were used in this cross-sectional analysis, which women with 24–30 weeks of gestation were included. Dietary folate intake was measured and their status was categorized by national guidelines. Methyl-donor nutritional quality index (MNQI) was used as a comprehensive indicator to assess the intake of folate and corelative nutrients in one-carbon metabolism. Binary logistic regression analysis was used to investigate the associations between dietary folate intake and GDM.

**Results:**

Among the 368 pregnant women included, 147 women (39.9%) were diagnosed with GDM. The proportion of participants with insufficient dietary folate intake (< 520 μg DFE/d) was 57.5%. While no significant association was observed between dietary folate intake and GDM, the highest odds of GDM was observed in women with combined insufficient vitamin B_12_ intake and folate intake in the highest quartile (*p* < 0.05).

**Discussion:**

In Conclusion, insufficient dietary folate intake in Chinese pregnant women during the second and third trimesters is rather concerning. It points out an urge for improving the dietary folate intake among pregnant women during this period. It also suggests a potential association between balanced vitamin B_12_ and folate intake and a lower likelihood of GDM.

## Highlights

- The dietary folate intake in Chinese pregnant women during the second and third trimesters is rather concerning.
- The results of binary logistic regression indicated that the intakes of folate, vitamins B_12_, energy, fat, and protein, and MNQI score were not significantly associated with GDM.
- When investigating combinations of folate and vitamin B_12_ intake status within the study population, we observed the highest odds of GDM for women with combined insufficient vitamin B_12_ intake and folate in the highest quartile.

## 1 Introduction

Gestational diabetes mellitus (GDM) is defined as glucose intolerance with onset or first recognition during pregnancy (1). If not properly managed, GDM may lead to adverse outcomes such as pre-eclampsia, preterm birth, still birth and cesarean birth (2, 3). Although GDM normally resolves after delivery, its effects may remain beyond pregnancy. Studies have shown that GDM patients are at higher risks of developing type 2 diabetes mellitus and cardiovascular diseases later in life, while their offspring are subject to obesity and diabetes at a later age (2, 4, 5). GDM is a common complication during pregnancy, affecting nearly 12.9% of pregnancies globally (6). In the Chinese mainland, the number is estimated to be 14.8% (7). Identifying modifiable nutritional factors associated with GDM is therefore of great public health importance. Recent studies have emphasized the interaction between nutritional and metabolic determinants in GDM development, supporting the concept that micronutrient imbalances may contribute to metabolic dysregulation during pregnancy (8).

Folate is an essential micronutrient in the synthesis of DNA, protein and lipids (9). It is known for its role in preventing neural tube defects in fetus (10). Since folate deficiency during gestation may cause adverse implications on fetal development and birthweight, supplementation with folic acid (the synthetic form of folate) is widely recommended by public health agencies around the world (11). Due to the critical role of folate in metabolism, researchers get interested in its association with GDM. Recent evidence suggested that maternal folate levels in early pregnancy were associated with alterations in glucose metabolism, although the relationship remained inconclusive, providing new insight into the metabolic link between folate and gestational glycemic control (12).

Dietary folate intake includes food folate and folic acid supplements. Many studies have investigated the effect of folic acid supplements on the risk of GDM, but food folate has received much less attention (7). A cohort study came to the conclusion that daily folic acid supplement intake in early pregnancy elevated the risk of gestational diabetes (13). Similarly, in a prospective cohort study among 4,353 Chinese pregnant women, it was observed that higher GDM risk was associated with folic acid supplement use of more than 800 μg/day from pre- to mid-pregnancy (14). Based on a birth cohort including 950 mother-offspring pairs from Hunan, China, folic acid supplementation for more than three months prior to pregnancy was found to be associated with a higher risk of gestational diabetes (adjusted relative risk (aRR): 1.72; 95% CI: 1.17–2.53) (15). A meta-analysis of cohort studies found that periconceptional folate exposure was positively associated with GDM, particularly among Chinese women (16). Consistent with these findings, recent studies reported that excessive plasma folate concentrations might disturb one-carbon metabolism and increase GDM risk. Conversely, in some prospective studies conducted in other populations, higher pre-pregnancy folate levels or moderate supplementation were inversely associated with GDM risk: In the Nurses’ Health Study II, which included 14,533 women, it was found that pre-pregnancy folic acid supplementation is linked to a decreased risk of gestational diabetes (17). Nevertheless, a Canadian investigation found no association between folic acid supplementation in early pregnancy and the risk of GDM (18). To explore the influence of both the duration and dose of folic acid supplementation before and during pregnancy, Chinese researchers conducted a case–control study with 1,300 pregnant women, using a self-report questionnaire at enrolment (19). They found a U-shaped association between folic acid supplementation and GDM risk, meaning that both insufficient and excessive folate intake could be risk factors.

Folate also acts as a carbon donor (also known as methyl donor) in one-carbon metabolism (OCM) (20). One-carbon metabolism is a set of biochemical reactions that transfer one-carbon units for various purposes, such as nucleotide synthesis, DNA methylation (21), amino acid homeostasis, and redox defense (22). These multiple functions make folate and other OCM nutrients essential for cell growth and survival, which is the foundation of pregnancy and fetal development. Therefore, some researchers started to factor other nutrients into the investigation of the association between folate and GDM. Though many observational studies have reported a linkage between one-carbon metabolic nutrients (especially folate and vitamin B_12_) and GDM, the underlying mechanism is still unclear (23–25). A possible explanation is that imbalances in OCM nutrients could influence insulin sensitivity or *β*-cell function via epigenetic regulation (e.g., DNA methylation) and oxidative stress (21, 26). Recent mechanistic and epigenetic studies have also demonstrated that methyl-donor nutrients, including folate and vitamin B_12_, modulate placental DNA methylation patterns and glucose metabolism in GDM pregnancies (27). To better integrate the combined effect of methyl-donor nutrients, Li et al. recently proposed the methyl-donor nutritional quality index (MNQI), which includes folate, choline, protein, vitamin B_12_, vitamin B_2_, vitamin B_6_ and zinc (28). This index enables a comprehensive assessment of methyl-donor nutrient quality in diet, it better reflects the overall methylation capacity than folate intake alone. Therefore, further exploration of the association between folate intake and GDM using the MNQI is warranted to provide a more comprehensive understanding of the role of folate in GDM outcomes.

To sum up, there is a lack of a comprehensive study to investigate the association of folate with GDM considering other nutrients relevant to folate metabolism. Therefore, this study is aimed to determine the association of dietary folate intake with GDM by adopting the MNQI to assess the dietary quality of pregnant women especially for methyl-donor nutrients (including folate, choline, protein, vitamin B_12_, vitamin B_2_, vitamin B_6_ and zinc), as well as to determine the association of dietary folate intake and corelative nutrients (vitamin B_12_ and homocysteine) of folate with GDM outcomes.

## 2 Methodology

### 2.1 Participants

This study was based on baseline data from the Mother & Child Nutrition and Health Cohort Study (MCNHC), a multicenter prospective cohort that follows mother–child pairs from mid-gestation through 7 years postpartum across five cities in China (Beijing, Gaomi, Changsha, Zhuzhou, and Qingdao).

The source population consisted of pregnant women receiving routine antenatal care in maternal and child healthcare hospitals in these cities. The study population included women who volunteered to participate in the MCNHC during their antenatal examination visits between September 2020 and June 2022, and who met the following inclusion criteria: (a) Pregnant women between the 24th and 32nd weeks of gestation; (b) Carrying a singleton pregnancy; and (c) Residing in the local area for a minimum of 5 years. Participants were selected through consecutive sampling, until the target of *n* = 200 (for each site) was reached. Pregnant women who met any of the following exclusion criteria were not included: (a) Using anti-folate medications; (b) Experiencing significant gestational complications; or (c) Demonstrating abnormal fetal intrauterine development. In the present study, the study unit was the individual pregnant woman. Details of the cohort establishment, including recruitment strategies and ethical approval, have been published elsewhere (29).

The sample size was initially estimated based on an expected GDM prevalence of approximately 15% among Chinese pregnant women with a 95% confidence level and a 5% margin of error, indicating that approximately 196 participants would be sufficient for prevalence estimation. After excluding withdrawals and those with missing key measurements, the final analytic sample comprised 368 pregnant women, which meets the estimated sample size and thereby ensures the reliability and statistical power of the study. No data imputation was performed.

The study was approved by the Peking University Institutional Review Board (Beijing, China, approval number: IRB00001052-19145) and adhered to the principles outlined in the Declaration of Helsinki. Written informed consent was procured from all pregnant participants, ensuring the safeguarding of their privacy and the confidentiality of their personal information, as well as that of their offspring.

### 2.2 Measurements and data collection

#### 2.2.1 Diagnosis of gestational diabetes mellitus

All pregnant women involved in the study were mandated to undergo a regular oral glucose tolerance test (OGTT) from 24 to 28 weeks of gestation in order to confirm the diagnosis of GDM, in accordance with the National Health Commission of China’s guidelines. GDM was diagnosed as a fasting blood glucose level 5.1 mmol/L, and/or 1 h post-OGTT level ≥ 10.0 mmol/L and/or 2 h post-OGTT ≥ 8.5 mmol/L as recommended by Chinese Medical Association and Chinese Society of Perinatal Medicine (30).

#### 2.2.2 Socio-demographic characteristics and GDM related factors of the participants

General information about the participants was obtained by self-administrated questionnaire. Details included socio-demographic characteristics (age, education level, household income), parity and lifestyle (pre-pregnant smoking exposure, pre-pregnant alcohol consumption, exercise). Body mass index (BMI) before pregnancy was collected by Physical and Health Status Questionnaire and calculated by dividing the weight in kilograms by the square of the height in meters.

#### 2.2.3 Dietary intake of folate and related nutrients

A comprehensive dietary survey was undertaken using the 24-h dietary record method for three consecutive days, comprising two weekdays and one weekend day, to ensure a holistic representation of dietary habits. The data collection process was facilitated through an online platform, Boohee, for seamless integration and accessibility. Prior to the survey, field personnel distributed food scales to the participating pregnant women and conducted training sessions on how to record their dietary intake accurately. During the recording period, pregnant women were consistently reminded to diligently document their meals on a daily basis, ensuring the integrity and reliability of the collected data.

The daily average intake of energy and nutrients, including carbohydrate, protein, fat, folate and its metabolically related nutrients (vitamins B_12_, B_6_, B_2_, choline and zinc), was calculated utilizing the comprehensive database sourced from the China Food Composition (31), ensuring accurate and reliable estimates of nutrients intake. The dietary intake of energy and nutrients were compared with the Chinese Dietary Reference Intakes (46). Inadequate intake was identified when the consumption of protein, folate, vitamin B_12_, B_2_, B_6_ and zinc fell below the estimated average requirement (EAR), whereas excessive intake was noted when exceeding the upper limit (UL). Additionally, dietary energy intake was evaluated using estimated energy requirements (EER, ±2SD), and the percentage of energy (%E) derived from carbohydrate and fat was compared against the acceptable macronutrient distribution ranges (AMDR).

#### 2.2.4 Methyl-donor nutritional quality index

Furthermore, we utilized the methyl-donor nutritional quality index (MNQI), a dietary quality index developed by our team (28), to thoroughly assess the consumption of folate and its metabolically associated nutrients (protein, vitamin B_2_, B_6_, B_12_, zinc, and choline). For above nutrient, a daily average intake within the range of 2/3 of the recommended nutrient intake (RNI) to the UL is scored as 2 points (protein, folate, and choline) or 1 point (vitamin B_2_, B_6_, B_12_, and zinc), whereas intakes below or above this range are scored as 0 points. The total score of MNQI ranges from 0 ~ 10, and the higher the score, the higher the dietary quality of folate metabolism. An MNQI score of 5 or below is indicative of poor dietary quality in terms of folate metabolism, whereas an MNQI score of 6 or above signifies better dietary quality for folate metabolism.

#### 2.2.5 Blood concentrations of folate metabolic indexes

Fasting venous blood samples of pregnant women at 24–32 gestational weeks were collected to measure red blood cell folate, serum folate, B_12_ and total homocysteine levels (tHcy), as detailed in the literature (29). The concentrations of red blood cell folate, serum folate, tHcy, and vitamin B_12_ were valid for 365 participants. These sample sizes were deemed sufficient for drawing scientifically valid conclusions, as supported by relevant literatures (32–34).

The folate deficiency was identified based on World Health Organization (WHO) standards (35), characterized by RBC folate levels below 151 ng/mL and/or serum folate concentrations less than 10 nmol/L. Vitamin B_12_ deficiency, on the other hand, was defined as a serum concentration of ≤148 pmol/L (36). Additionally, hyperhomocysteinemia (HHcy) was categorized as tHcy levels equal to or exceeding 10 μmol/L (37).

### 2.3 Statistical analysis

Descriptive data were presented as frequencies and percentages for categorical variables, while means and standard deviations (SD) were generated for continuous variables. To compare the characteristics between the GDM group and the non-GDM group, we conducted the Student’s *t*-test for continuous variables and Pearson’s Chi-squared test for categorical variables. Differences in concentrations across groups were compared using non-parametric analysis. The Wilcoxon rank-sum was adopted as the distributions for dietary intake were skewed. Logistic regression models were performed to analyze the associations between multiple factors and GDM odds. The events-per-variable (EPV) ratio was assessed to ensure adequate model stability. With 147 GDM cases, all multivariable logistic regression models satisfied the commonly recommended ≥ 10 EPV rule. To account for multiple comparisons, Bonferroni or false discovery rate (FDR) were used for correction. FDR (adjusted q-values) were calculated using the Benjamini-Hochberg procedure within each family of related tests. In this study, statistical analyses were performed on SPSS Version 20.0 (IBM Corp., Armonk, N.Y., United States). *p* values less than 0.05 were considered statistically significant.

## 3 Results

### 3.1 Characteristics of the participants

A total of 368 pregnant women were included in the present study, of whom 147 (39.9%) had GDM. The mean (± SD) age of the study population was 33.08 (± 4.64) years. Most of the pregnant women (86.7%) were in their second trimester (between 14 and 27 gestational weeks).

The characteristics of the study population by GDM status are summarized in Table 1. Age ≥35 years old was significantly related to a higher GDM odds than age <35 years old (*p* < 0.05). Pregnant women with sufficient physical activity (≥30 min/d) had significantly a lower odds of GDM than those with insufficient physical activity (<30 min/d) did (*p* < 0.05). Due to the voluntary enrollment, among the total of 368 participants with GDM diagnosis, not every participant had information collected on socio-demographic features (332 available), dietary intake (334 available) and blood concentrations (365 available). The missing data were not imputed.

**Table 1:** Characteristics of the participants.

Characteristics of pregnant women	Total	Gestational diabetes mellitus (GDM)	*P* value
Total	368 (100.0)	147 (39.9)	
Age (years)^a^			0.031*
<35	215 (63.2)	79 (36.7)	
≥ 35	125 (36.8)	61 (48.8)	
Gestation (weeks)			0.166
24 ~ 27	319 (86.7)	123 (38.6)	
28 ~ 30	49 (13.3)	24 (49.0)	
Education^a^			0.951
Below college	99 (29.8)	42 (42.4)	
College and above	233 (70.2)	98 (42.1)	
Household income (per capita monthly, yuan)^a^			0.326
<5,000	179 (55.6)	68 (39.1)	
≥5,000	139 (44.4)	62 (44.6)	
Pre-pregnant BMI (kg/m^2^)^a^			0.267
Wasted (<18.5)	27 (7.8)	8 (29.6)	
Normal (18.5 ~ 23.9)	193 (56.1)	77 (39.9)	
Overweight (24.0 ~ 27.9)	83 (24.1)	38 (45.8)	
Obesity (≥28.0)	41 (11.9)	21 (51.2)	
Pre-pregnant smoking exposure^a^			0.374
No	190 (62.1)	82 (43.2)	
Yes	116 (37.9)	44 (37.9)	
Pre-pregnant alcohol consumption^a^			0.329
No	276 (90.2)	111 (40.2)	
Yes	30 (9.8)	15 (50.0)	
Physical activity^a^			0.049*
Sufficient (≥30 min/d)	171 (55.9)	62 (36.3)	
Insufficient (<30 min/d)	135 (44.1)	64 (47.4)	
Parity^a^			0.895
Nulliparous	189 (55.4)	77 (40.7)	
Multiparous	152 (44.6)	63 (41.4)	

Data were presented as *n* (%). Pearson χ^2^ test was used to compare the categorical variables. * *p* < 0.05.

a Variables have missing data. Missing data were not imputed.

### 3.2 Association between dietary intake of folate and related nutrients and GDM outcomes

By summarizing the dietary intakes of folate and other one-carbon metabolism related nutrients among the study subjects, the proportion of insufficient folate intake (< EAR) among participants was 52.2, and 19% of the participants had excessive folate intake (≥ UL). 48.8% of the participants consumed vitamin B_12_ less than its EAR. The MNQI score ranged from 0 to 8 points with a median of 5 points.

The results of binary logistic regression indicated that dietary intake of folate and other one-carbon metabolism related nutrients involved in the MNQI were not significantly associated with GDM. Adequate carbohydrate intake (50% ~ 65%) was associated with a lower odds of GDM after adjusting for age, gestational age, education, income, physical activity, pre-pregnancy BMI, parity and dietary energy intake (adjusted OR = 0.46, 95% CI: 0.27 to 0.78, *p* < 0.05) (as shown in Table 2). After controlling the false discovery rate, the overall pattern of associations remained similar, with carbohydrate intake showing the lowest q-value and other nutrients remaining non-significant.

**Table 2:** Associations between of dietary intake of folate and other one-carbon metabolism related nutrients with gestational diabetes mellitus (GDM).

Dietary intake	*n* (%)	GDM	*P*
		*ORs* (95% *CI*)	
Folate			
EAR~RNI	21 (5.7)	Reference	
<EAR	192 (52.2)	0.93 (0.33, 2.63)	0.892
RNI ~ UL	51 (13.9)	0.55 (0.17, 1.82)	0.327
≥UL	70 (19.0)	0.95 (0.31, 2.92)	0.924
Vitamin B_12_			
<EAR	163 (48.8)	Reference	
≥EAR	171 (51.2)	1.12 (0.67, 1.86)	0.679
Protein			
<EAR	78 (23.0)	Reference	
≥EAR	261 (77.0)	1.31 (0.68, 2.54)	0.422
Vitamin B_2_			
<EAR	150 (44.9)	Reference	
≥EAR	184 (55.1)	0.77 (0.46, 1.27)	0.303
Vitamin B_6_			
<EAR	241 (72.2)	Reference	
≥EAR	93 (27.8)	0.88 (0.50, 1.55)	0.667
Zinc			
<EAR	103 (30.8)	Reference	
≥EAR	229 (68.6)	1.28 (0.73, 2.22)	0.391
MNQI score			
<5	149 (44.5)	Reference	
≥5	186 (55.5)	0.74 (0.45, 1.22)	0.240
Energy			
Sufficient (EER ± 2SD)	109 (32.5)	Reference	
Insufficient (<EER−2SD)	194 (57.9)	0.91 (0.53, 1.57)	0.739
Excessive (≥EER + 2SD)	32 (9.6)	0.93 (0.38, 2.27)	0.874
Carbohydrate (%E)			
<50%	126 (37.6)	Reference	
50% ~ 65%	183 (54.6)	0.46 (0.27, 0.78)	0.004*
≥65%	26 (7.8)	0.47 (0.17, 1.34)	0.160
Fat (%E)			
<20%	23 (6.9)	Reference	
20% ~ 30%	130 (38.8)	1.59 (0.52, 4.81)	0.415
≥30%	182 (54.3)	2.11 (0.71, 6.29)	0.182

Models were adjusted for age, gestational weeks, education, income, physical activity, pre-pregnancy BMI and parity, dietary energy. * *P* < 0.05. The percentages were valid percentages.

### 3.3 Combination of dietary folate and vitamin B_12_ intake with GDM

The highest OR of GDM was observed in the participants with combined dietary folate intake in the highest quartile and insufficient vitamin B_12_ intake, compared with women with insufficient intake of vitamin B_12_ but folate intake in the lowest quartile (*p* < 0.05). In contrast, no significant associations between higher folate intake and GDM were found among women with sufficient vitamin B_12_ intake (Table 3).

**Table 3:** Associations between combinations of maternal vitamin B_12_ intake and folate intake, and GDM.

Dietary intake	B_12_-insufficient (< EAR)		B_12_-sufficient (≥ EAR)	
	*ORs* (95% *CI*)	*P*	*ORs* (95% *CI*)	*P*
Folate				
Q1	Reference		Reference	
Q2	1.18 (0.50, 2.82)	0.705	1.51 (0.30, 7.53)	0.617
Q3	0.37 (0.13, 1.12)	0.079	0.43 (0.10, 1.92)	0.266
Q4	17.50 (1.72, 177.80)	0.016*	0.48 (0.12, 2.00)	0.314

Q, quartile. Models were adjusted for age, gestational weeks, education, income, physical activity, pre-pregnancy BMI, parity, and dietary energy. * *P* < 0.05.

The logistic regression model further confirmed a significant interaction between dietary folate intake and vitamin B_12_ sufficiency (Wald χ^2^ = 9.27, *p* = 0.026), supporting that the effect of folate on GDM was dependent on maternal vitamin B_12_ status. A sensitivity analysis further adjusting for folic acid supplementation yielded similar results overall, although the significant association shifted from Q4 to Q3, suggesting partial confounding by supplement use.

### 3.4 Association between blood concentrations of folate metabolic indexes and the odds of GDM in pregnant women

Blood concentrations of folate, vitamin B_12_ and homocysteine were divided into quartiles based on their distribution cutoff points in the study population, and the lowest quartile was used as a reference. Although RBC folate in Q3 was significantly associated with GDM in the unadjusted model, the results indicated no significant association between RBC folate, serum folate and serum homocysteine neither in total nor in quartiles with GDM, after adjusting for maternal age, education, monthly household income per capita, pre-pregnant BMI, physical activity, parity, and dietary energy (Table 4). The distribution of GDM cases across quartiles ensured sufficient events per variable, with all models meeting the ≥ 10 EPV criterion.

**Table 4:** Associations between blood folic acid, vitamin B_12_ and homocysteine concentrations with GDM.

Blood concentration	Unadjusted	Model 1	Model 2
	OR (95%CI)	OR (95%CI)	OR (95%CI)
RBC folate (ng/mL)^a^			
Q1 (<726.29)	Reference	Reference	Reference
Q2 (726.29 ~ 1017.77)	1.70 (0.94, 3.08)	1.14 (0.58, 2.26)	1.15 (0.57, 2.29)
Q3 (1017.77 ~ 1181.30)	1.89 (1.04, 3.44)*	1.59 (0.78, 3.26)	1.57 (0.75, 3.26)
Q4 (>1181.30)	0.86 (0.46, 1.60)	0.66 (0.31, 1.37)	0.62 (0.29, 1.32)
Total	0.97 (0.81, 1.17)	0.91 (0.73, 1.15)	0.90 (0.71, 1.13)
Serum folate (ng/mL)^b^			
Q1 (<8.92)	Reference	Reference	Reference
Q2 (8.92 ~ 13.92)	1.23 (0.68, 2.21)	1.38 (0.70, 2.72)	1.36 (0.68, 2.71)
Q3 (13.92 ~ 19.91)	1.31 (0.73, 2.36)	1.07 (0.53, 2.14)	0.98 (0.48, 2.03)
Q4 (>19.91)	0.68 (0.37, 1.25)	0.77 (0.37, 1.62)	0.71 (0.33, 1.52)
Total	0.90 (0.75, 1.09)	0.91 (0.72, 1.14)	0.88 (0.69, 1.11)
Serum vitamin B_12_ (pmol/L)^c^			
Q1 (<187.50)	Reference	Reference	Reference
Q2 (187.50 ~ 227.00)	0.80 (0.44, 1.46)	0.87 (0.43, 1.79)	0.94 (0.45, 1.97)
Q3 (227.00 ~ 270.00)	1.34 (0.74, 2.41)	1.45 (0.72, 2.90)	1.71 (0.82, 3.55)
Q4 (> 270.00)	1.05 (0.58, 1.89)	1.03 (0.51, 2.11)	1.10 (0.52, 2.33)
Total	1.07 (0.89, 1.29)	1.07 (0.85, 1.33)	1.09 (0.86, 1.39)
Serum total homocysteine (μmol/L)^d^			
Q1 (<3.90)	Reference	Reference	Reference
Q2 (3.90 ~ 4.80)	1.08 (0.59, 1.96)	0.78 (0.37, 1.65)	0.89 (0.41, 1.94)
Q3 (4.80 ~ 5.56)	1.19 (0.65, 2.17)	0.79 (0.38, 1.63)	0.83 (0.39, 1.77)
Q4 (>5.56)	1.73 (0.95, 3.15)	1.12 (0.55, 2.30)	1.25 (0.59, 2.67)
Total	1.19 (0.99, 1.44)	1.05 (0.83, 1.32)	1.07 (0.84, 1.36)

Q, quartile. Model 1 was adjusted for age, gestational age, education, income, physical activity, pre-pregnant BMI, parity, and dietary energy.

a In Model 2, vitamins B_12_ was adjusted on the basis of Model 1.

b In Model 2, vitamins B_12_ was adjusted on the basis of Model 1.

c In Model 2, RBC folate and serum folate were adjusted on the basis of Model 1.

d In Model 2, RBC folate, serum folate and vitamins B_12_ were adjusted on the basis of Model 1.

## 4 Discussion

### 4.1 Dietary folate intake among Chinese pregnant women

Gestational diabetes mellitus (GDM) is a common pregnancy complications that may lead to short-term and long-term impact on the mothers and their offspring. The relatively higher prevalence rate of gestational diabetes mellitus (GDM) observed in this study (39.9%) was partially due to the fact that the study population was not a natural one, as some of the participants were referred from nutrition department. It probably also resulted from an increasing trend in GDM incidence globally (38) and within China (7). This upward trajectory is driven by demographic and lifestyle shifts, including rising rates of advanced maternal age, obesity, and sedentary urban lifestyles (39, 40). Moreover, the urban environment of the collaborating local maternal and child healthcare clinics plays a critical role. For example, studies in China have shown a higher incidence of GDM among urban, older, and more highly educated pregnant women (41). The specialized institutions in this study might attract a patient demographic that already has a higher baseline prevalence of GDM risk factors.

Known GDM risk factors include advanced maternal age, obesity and lack of exercise (42). Ethnicity, genetics, and lifestyle other than exercise could also contribute to the disorder (43). In this study, advanced maternal age (≥35 years) and insufficient physical activity (<30 min/d) were found to be potentially associated with GDM, consistent with previous research findings. These findings further underscore the importance of giving birth at an appropriate maternal age and engaging in adequate physical activity during pregnancy. They also suggest that maternal and child health institutions should enhance health education to raise awareness of modifiable risk factors for GDM.

Several studies have addressed the relationship between dietary folate intake and GDM, however, significant association was not found in our study. Differences in study design, population characteristics and dietary assessment methods may explain the inconsistent associations observed between folate intake and GDM outcomes. While preconception folic acid supplementation was found to be linked with a reduced GDM risk by Li et al. (17), another large-sample study showed opposite finding that consuming folic acid supplementation more than 800 μg/day from pre-pregnancy through mid-pregnancy significantly increased GDM risk (14). Nevertheless, a Canadian survey found a null correlation between folic acid supplementation in the early pregnancy and risk of GDM (18). But these studies focused on folate intake before or in early stage of pregnancy, in our study, dietary folate intake was assessed in the second or third trimester. Our observation that folate intake was not significantly associated with GDM risk is supported by the China-Anhui Birth Cohort Study (13). Although Zhu et al. (13) found that taking folic acid supplement daily during the first trimester would increase the risk of GDM, folic acid supplementation in the second trimester did not appear to influence GDM risk. Still, most of previous studies only investigated folic acid supplementation by questionnaires (13, 14, 17), while our study also employed the 3-day 24-h dietary record together with a questionnaire, in order to assess the dietary folate intake both from food and supplementation in a more accurate manner. More data are needed to understand the relationship between total dietary folate intake and GDM risk.

Interestingly, we found that participants with insufficient intake of vitamin B_12_ and folate in the highest quartile had the highest OR of GDM compared to those with insufficient intake of vitamin B_12_ but folate intake in the lowest quartile. However, there were no significant associations between higher folate intake and GDM among women with sufficient vitamin B_12_ intake. To our knowledge, no published study has examined the combination of consumption status of folate and vitamin B_12_ to explore their association with GDM. Previous studies had observed a higher odds of GDM with the combination of high folate and low vitamin B_12_, but what they measured were the blood concentrations of the two vitamin Bs (24, 44, 45). A potential biological explanation is that concurrent insufficiency of folate and vitamin B_12_ may impair one-carbon metabolism, resulting in oxidative stress, and impaired insulin signaling, which could contribute to the development of GDM (21, 26). Our findings could provide some evidence in favor of the hypothesis that maintaining adequate levels of both folate and vitamin B_12_ is beneficial for reducing the likelihood of gestational diabetes mellitus. They suggest implications for promoting optimal nutrition in pregnant women and improving maternal and offspring health.

The strengths of this study included combining dietary intake, blood concentration of folate and relative nutrients and the comprehensive indicator MNQI to explore the relationship between folate metabolism and GDM, providing a multidimensional assessment of folate status. Additionally, folate and other nutrients related to folate metabolism were investigated in a single laboratory using the same instruments and settings minimizing test-induced variability. Finally, some available confounding factors were adjusted in the statistical analysis. However, several limitations should be mentioned. First, as a cross-sectional study, causal relationships between folate or vitamin B_12_ intake and GDM cannot be established. Second, the study population was limited to two centers in Beijing and Shandong, which may restrict the generalizability of the findings to broader populations. Regional and ethnic variations in nutrient metabolism further underscore the need for future multicenter studies. Given the unique dietary patterns and fertility culture in Chinese pregnant women, these results may have population-specific implications. Third, dietary intake was assessed using self-reported 3-day food records, which may be subject to recall and reporting bias despite standardized data collection procedures. Finally, certain established risk factors for GDM, such as detailed family history of diabetes and genetic profiles, were not comprehensively assessed due to constraints in the original cohort design and limited sample size for some variables. Future studies should incorporate these factors to allow more in-depth analysis. In conclusion, our study examined folate intake and other nutritional factors during pregnancy and suggested that the intakes of folate, vitamin B_12_, energy, fat, and protein, and MNQI score were not significantly associated with GDM. The highest odds of GDM was observed in women with combined insufficient vitamin B_12_ intake and folate in the highest quartile, indicating the association between imbalance between folate and vitamin B_12_ and GDM. Future research should include prospective cohort and intervention studies investigating the combined effects of folate and vitamin B_12_ supplementation on GDM outcomes in ethnically and geographically diverse populations.

## Data Availability

The raw data supporting the conclusions of this article will be made available by the authors, without undue reservation.
